# Interprofessional communication skills training to improve medical students’ and nursing trainees’ error communication - quasi-experimental pilot study

**DOI:** 10.1186/s12909-023-04997-5

**Published:** 2024-01-03

**Authors:** Lina Heier, Barbara Schellenberger, Anna Schippers, Sebastian Nies, Franziska Geiser, Nicole Ernstmann

**Affiliations:** 1 Center for Health Communication and Health Services Research, Department of Psychosomatic Medicine and Psychotherapy, University Hospital Bonn, Bonn, Germany; 2 Chair of Health Services Research, Institute of Medical Sociology, Health Services Research, and Rehabilitation Science, Faculty of Medicine and University Hospital Cologne, University of Cologne, Cologne, Germany; 3 Department of Clinical Pharmacy and Toxicology, Maastricht University Medical Center, Maastricht, The Netherlands; 4 CARIM School for Cardiovascular Disease, Maastricht University, Maastricht, The Netherlands; 5 Centrum für Aus- & Weiterbildung, University Hospital Bonn, Bonn, Germany; 6 Department of Psychosomatic Medicine and Psychotherapy, University Hospital Bonn, Bonn, Germany

**Keywords:** Communication skills training, Interprofessional training, Pre-post design, Medical education, Nursing education, Mixed-methods, Error communication, Simulation

## Abstract

**Background:**

Interprofessional communication is of extraordinary importance for patient safety. To improve interprofessional communication, joint training of the different healthcare professions is required in order to achieve the goal of effective teamwork and interprofessional care. The aim of this pilot study was to develop and evaluate a joint training concept for nursing trainees and medical students in Germany to improve medication error communication.

**Methods:**

We used a mixed-methods, quasi-experimental study with a pre-post design and two study arms. This study compares medical students (3rd year) and nursing trainees (2nd year) who received an interprofessional communication skills training with simulation persons (intervention group, IG) with a control group (CG). Both cohorts completed identical pre- and post-training surveys using the German Interprofessional Attitudes Scale (G-IPAS) and a self-developed interprofessional error communication scale. Descriptive statistics, Mann-Whitney-U-test and Wilcoxon-test were performed to explore changes in interprofessional error communication.

**Results:**

A total of 154 were medical students, and 67 were nursing trainees (IG: 66 medical students, 28 nursing trainees / CG: 88 medical students, 39 nursing trainees). After training, there were significant improvements observed in the “interprofessional error communication” scale (p < .001) and the “teamwork, roles, and responsibilities” subscale (p = .012). Median scores of the subscale “patient-centeredness” were similar in both groups and remained unchanged after training (median = 4.0 in IG and CG).

**Conclusions:**

Future studies are needed to find out whether the training sustainably improves interprofessional teamwork regarding error communication in acute care.

## Background

Harm caused by medication errors is described as adverse drug event (ADE) and errors can range from minor with no harm to major errors with serious harm and death [1]. ADE happen on a regular basis and are part of the routine care: a systematic review reports an occurrence of ADEs in surgical patients between 2.0 and 27.7 of 100 admissions or 8.9% of the patients [2]. Elliott et al. (2021) have estimated that 237.3 million medication errors happen annually in England and occur at all stages and settings where medication is in use [3]. An estimated 15–59% of ADEs are considered as preventable [4]. An open und trustful communication about medication errors is an important part of the responsibilities of healthcare professionals (HCPs). It is essential to limit further potentially harmful consequences of an error for both the patient with his family and the team, and it is necessary in order to prevent future errors. It requires skills, attitudes and knowledge to discuss medication errors in an adequate way with patients and their families, supervisors and colleagues [5]. HCPs need to be trained in error communication to fulfil the requirements of a professional communication about it. Interventions to train interprofessional teams in terms of error communication and patient safety are already a fixed component of training in healthcare [6, 7] and interventions to improve (error) communication are rising [8–12].

The majority of students from different health disciplines (e.g., medicine, nursing, pharmacy) have a positive attitude towards IPE and collaborative work [13–16]. It can contribute to a better understanding of the professional role of the other profession, improve collaborative knowledge and skills, identify and solve patient problems and minimise medical errors [17, 18]. Benefits are also seen in better communication, teamwork or elimination of hierarchies [15–18]. Disadvantages contain lack of student engagement and information overload [15]. In a systematic review, perceptions of medical students, residents and nursing students regarding IPE in a clinical setting were analysed. From quantitative, qualitative and mixed-methods studies, expectations and attitudes were considered in the three categories of readiness, facilitators and barriers at the individual, process and cultural levels. At the individual level, readiness for IPE is higher among women and younger students, and students with previous healthcare experience [15, 19]. Enabling factors at the individual level are respect, trust and role clarity between professional groups. In contrast, emphasis on expertise, arrogance and lack of teamwork skills are barriers to IPE. At the process level, readiness for IPE varies during studies, a barrier is the lack of formal assessment, conducive is learning in small groups and in an authentic context. On a cultural level, IPE is difficult if the participants from the different professions do not know each other; getting to know each other informally can be supportive [19].

To introduce future HCPs to interprofessional work and communication at an early stage, interprofessional education initiatives have been in place for several years [20]. Interprofessional education (IPE) describes the learning with, from, and about two or more healthcare professions to improve collaboration and healthcare [21]. Recently, a systematic review analysed IPE about patient safety and medication errors and reported no IPE activity focusing on professional communication about medication errors [22]. IPE activities are concentrating on improving education in medication safety, interprofessional collaboration or/and medication use process [22].

This research gap in IPE is addressed by this paper: the first aim of the present pilot study was to develop a joint communication skills training for nursing trainees and medical students in professional error communication. Medical students and nursing trainees should learn to communicate errors in an interprofessional team already during their studies. The second aim was to examine the effectiveness, i.e. to test whether the intervention is associated with improvements in interprofessional attitudes and self-reported skills in interprofessional error communication. Our hypothesis is that the intervention group (IG) will show significantly better scores than the control group (CG) on the scales “teamwork, roles, and responsibilities”, “patient-centeredness”, and “interprofessional error communication” after the intervention. A third aim was to investigate the trainings’ acceptance, feasibility, and implementation conditions.

## Methods

### Study design

**Fig. 1:**
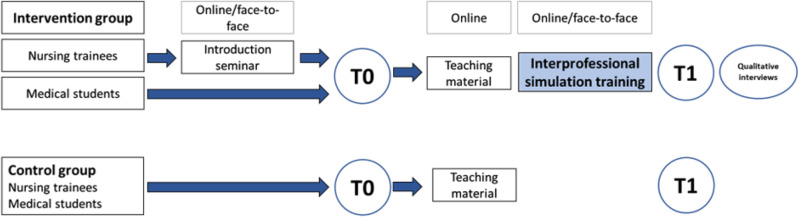
Study design

We present results from a quasi-experimental study with a pre-post, mixed-method design (convergent parallel design) and two study arms in which quantitative data were obtained from questionnaires at two points in time and qualitative data were obtained from semi-structured interviews. The study was conducted between October 2021 and February 2023 at one University Hospital in Germany. This study compares a cohort of medical students and nursing trainees who received an interprofessional communication skills training about medical errors in acute care (IG) with a cohort who did not receive the interprofessional training (CG). The IG completed a questionnaire before (T0) and immediately after the simulation talk (T1). The CG of medical students completed T0 before the uniprofessional communication simulation training and T1 immediately after. As the CG of the nurses did not receive simulation training, they completed T0 at an information session at the beginning of the study (T0) and T1 at an agreed appointment three months later. The study design is presented in detail in Fig. 1. The study was approved by the Ethics Committee of the Medical Faculty of the University of Bonn, Germany (Reference number for approval: 147/22).

### Participants and setting

Third-year medical students and nursing trainees in their first or second year of apprenticeship participated in the study. In Germany, registered nurses undergo a three-year training program at nursing schools, culminating in a state examination. Nursing schools that integrate the specialisation in acute care in their training are typically connected to hospitals, providing trainees with the opportunity to gain practical experience from the start of their program, supervised by experienced staff. Nursing trainees are responsible for providing physical care to patients, assisting team members, and offering guidance and support to patients and their families and are prepared for the responsible assumption of the supervision of the care process.

In order to implement the interprofessional communication skills training without changing the curriculum of both professions, it was embedded in the regular and mandatory course “Communication and Conversation Skills” for third-year medical students. Participating medical students are at the beginning of their clinical section of their studies and typically have clinical experience through initial clinical clerkships. The medical students of the IG took part in an interprofessional training, the CG in the regular course with uniprofessional simulation talks. In order to offer as many simulation talks as possible, all nursing trainees in their second year of apprenticeship were assigned into the IG and all nursing trainees in their first year of apprenticeship were assigned to the CG. Since there are more medical students in one semester at the University Hospital than in one year of apprenticeship of nursing, the IG is nevertheless smaller than the CG. Nursing trainees in their second year of apprenticeship participated in the communication training (IG). Since they did not regularly attend the course, they were given an information session on the procedure of the communication training. The CG of the nursing trainees in their first year of apprenticeship did not take part in any interprofessional training.

Inclusion criteria were (a) medical students (enrolled at the Rheinische Friedrichs-Wilhelms-University Bonn) in the clinical section in their third year, participating in the course “Communication and Conversation Skills”; (b) nursing trainees (enrolled at the university hospital in Bonn) in their first or second year of apprenticeship.

### Interprofessional communication skills training

In the regular course with uniprofessional communication skills training, a group consists of six medical students who have two appointments: on one appointment they conduct one of the three simulation talks, and on one appointment they observe the other interviews. For the interprofessional simulations, three nursing trainees per group took part additionally. In a 90-minute simulation session, three simulation talks with simulation persons were conducted. Participants had ten minutes to prepare, then ten minutes for the simulation talk followed by ten minutes of standardised 360° feedback from four perspectives (self-reflection, observers of the other professional group, simulation persons, and from a trainer). Students and trainees were able to agree in advance on the seating arrangement and the conversation (e.g., who will start the conversation).

The interprofessional communication skills training was developed by an interprofessional team of health services researchers, psychologists and registered nurses, all experienced in patient safety and clinical care. Based on scenarios reported in a critical incident reporting system (CIRS) in Germany, three different scenarios were developed, all focusing on medication errors. All scenarios were constructed in such a way that the adverse event was caused by a chain of errors. The students’ and trainees’ task was to communicate the error openly, honestly and fact-based and to endure the situation. Students and trainees were able to agree in advance on the seating arrangement and the conversation (e.g. who will start the conversation). One scenario deals with a medication error and the changing care for a patient, one situation deals with the explanation of a medication error with the medical supervisor and one situation revolves around a conversation with a relative about a medication error. In Table 1 an overview about the three scenarios is shown. Simulation persons supported communication skills training by acting as the patient, supervisor, or family member. In this way, the medical students and nursing trainees had a conversation with the actor together as an interprofessional team.

**Table 1:** Overview about the three scenarios

	Scenario 1	Scenario 2	Scenario 3
Error	Uncertainty about whether the right chemotherapy drug was given	Given the wrong antibiotic	Preparation of chemotherapy and patient due to interchanged laboratory values
Setting	In a physician’s room on the ward	In a physician’s room on the ward	In a physician’s room on the ward
Nursing trainee	Registered Nurse	Registered Nurse	Registered Nurse
Medical student	Assistant physician	Assistant physician	Assistant physician
Simulation person	Patient	Relative	Senior physician
Diagnosis	Colon cancer	Severe infection	Cancer

### Data collection

All participants (IG and CG) received online teaching materials and conversation guides on the topic of interprofessional error communication, resulting in a blended-learning format. The teaching material was provided online via the medical students’ regular teaching and learning platform. The nursing trainees were given their own access and room with the same material on this platform. Medical students received an email with the link to the online survey via the platform and their study number to track pre and post responses. The survey was open for three weeks with weekly reminders per email. As the nursing trainees did not regularly attend the course, they were asked to fill in the same questionnaire in paper form at a face-to-face meeting. The questionnaire included an informed consent explaining the purpose of the study, voluntariness of participation, and that by completing and submitting the questionnaire informed consent of participation was confirmed.

The second questionnaire was filled out by the participants directly after their simulation training. Since the nursing trainees of the CG did not take part in any simulation training, they completed the second questionnaire in paper form at a separate face-to-face-meeting.

### Measures and data analysis

IG and CG completed identical pre- and post-training surveys using the adapted German Interprofessional Attitudes Scale (G-IPAS) [23] and a self-developed interprofessional error communication scale. The adapted G-IPAS consists of two scales (“Teamwork, Roles and Responsibilities”, “Patient-centeredness”) and 17 items [23]. As we expected an improvement in the attitude towards interprofessional error communication and no suitable scale was found in the literature, an interprofessional expert group (psychology, nursing, public health, speech and language therapy, health services research), developed and pretested a scale. The self-developed scale “interprofessional error communication” was based on the formulation of the G-IPAS and consists of 4 items. All scales are using a five-point Likert scale (from 0 (strongly disagree) to 4 (strongly agree)). Additional items captured the demographic characteristics of the study participants.

Mean values and standard deviations for each scale were calculated. Cronbach`s alpha, as an indicator of internal consistency of the newly developed scale “interprofessional error communication”, was calculated. Mann-Whitney-U-test and Wilcoxon-test were performed to explore group differences between IG and CG. All tests applied a p = .05 level of significance. SPSS Version 27 was used for analysing.

### Formative evaluation

To investigate feasibility aspects and implementation conditions, participants from IG were interviewed about interprofessional learning and the course (teaching material and simulation talks) for formative evaluation. Two female researchers and a female student assistant with a background in psychology and health services research, conducted individual semi-structured interviews face-to face or via phone between one and three months after intervention. Each interview was audio-recorded, fully transcribed, anonymized, and coded using content analysis [24]. The researchers read the interviews, marked important text passages, developed, and defined codes inductively. Identified codes were used as coding system for the entire material. Text passages with the same main categories were compiled and subcategories were developed inductively. During analysis, anchor quotations were chosen. All findings were discussed in a multidisciplinary team of researchers.

## Results

### Participants characteristics

**Fig. 2:**
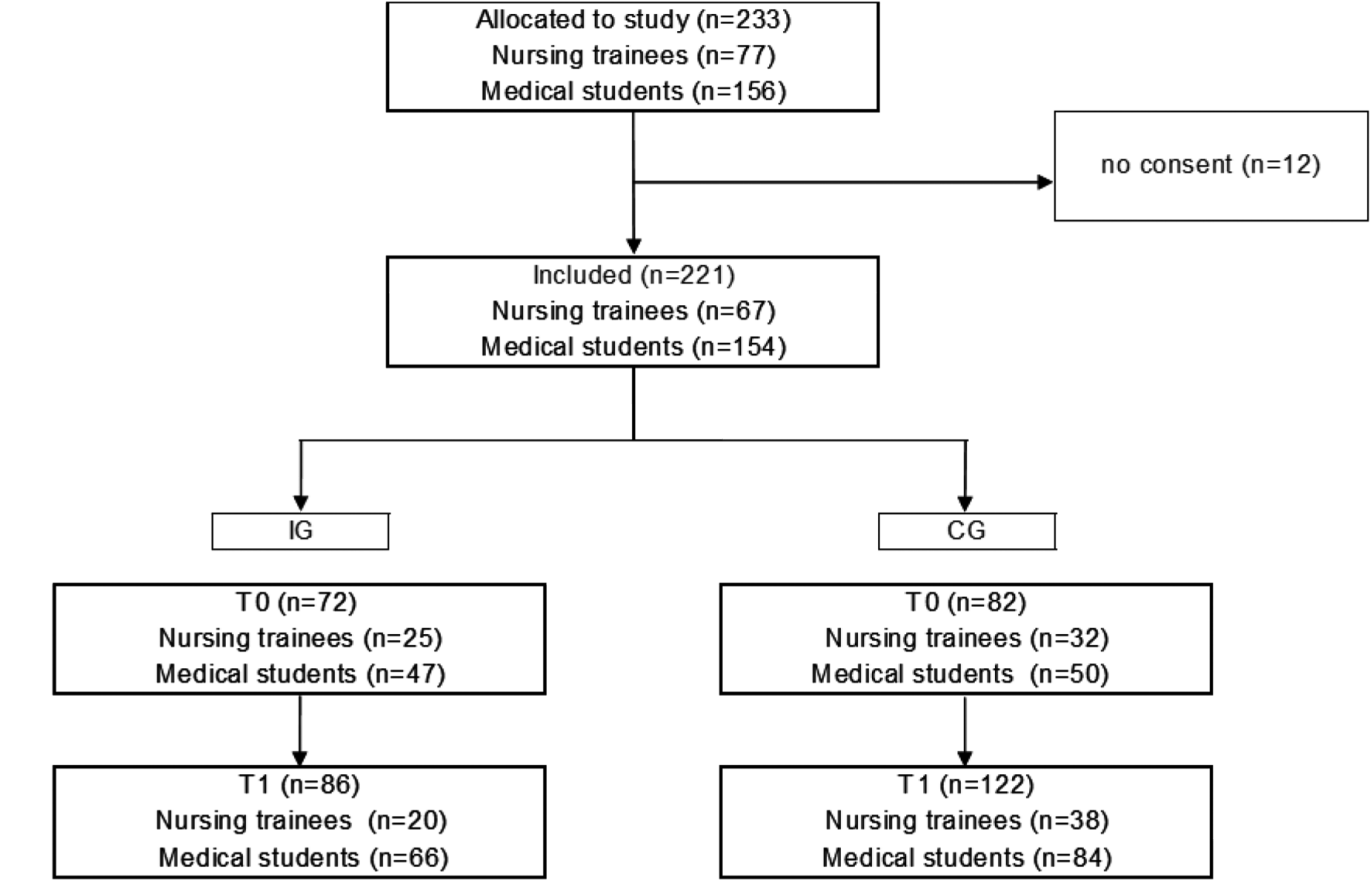
Flow chart of study participants

Of the 233 participants across both groups who were allocated to our study, 12 participants gave no consent for participation, resulting in N = 221 participants. 67 participants were nursing trainees (31.3%), 154 were medical students (69.7%). In some cases, only a few nursing trainees were present in the courses, as the course was not compulsory for them. In these cases, nursing trainees practised the roles several times or medical students took over the role of nurses. The participating nursing trainees in the IG all actively participated in the simulation talks. Due to the different group sizes (medical students, nursing trainees), there were medical students who conducted a simulation talk as well as those who only observed the simulation talks. Flow chart (Fig. 2) presents an overview of the allocation. Mean (SD) age was 24 (3.9) years, the youngest participant was 18 years old, the oldest was 36 years old. 113 participants identified as female, 34 as male and 2 as diverse. 48 participants have completed a previous vocational training program (21.7%).

### Descriptive statistics and training associations

Table 2 provides an overview of the descriptive statistics (n, mean, standard deviation and Cronbach’s alpha) for each scale at T0 (pre training) and T1 (post training) between IG and CG. Each subscale (α = 0.91–0.79) achieved acceptable levels of internal consistency. Mean score of the scales and of IG and CG was between 2.6 (CG at T0: interprofessional error communication) and 3.9 (IG at T0: patient-centeredness). All mean scores improved after training (T1).

To explore differences of the interprofessional communication skills training between IG and CG at T0 and T1, we used Mann-Whitney-U-Test, and to explore changes between T0 and T1 within the groups, we used Wilcoxon-Test (Table 2). After training, significant increases were observed in the self-developed scale “interprofessional error communication” (p < .001) and the subscale “teamwork, roles, and responsibilities” of G-IPAS (p = .012) at a significance level *p* < .05. Median scores of the subscale “patient-centeredness” did not change after training (median = 4.0 at T0 and T1 in IG and CG).

In IG, significant increases between T0 and T1 were observed in the scale “interprofessional error communication” and “teamwork, roles, and responsibilities”. In CG, a significant, albeit smaller increase was found only in the scale “interprofessional error communication”.

**Table 2:** Descriptive statistics for the three scales “interprofessional error communication”, “patient-centeredness” and “teamwork, roles and responsibilities”

		T0						T1							p-Value^a^
		N	Mean	SD	Median	IR	IG vs. CG p-Value^b^	N	Mean	SD	Median	IR	IG vs. CG p-Value^b^	Cronbachs Alpha	T0 vs. T1
Interprofessional error communication	IG	71	2.67	0.95	2.75	3.87–4.00		86	3.35	0.69	3.50	3.00–4.00			**< 0.001**
	CG	80	2.60	0.89	2.50	2.00–3.35		122	2.91	0.85	3.00	2.50–3.50			**0.001**
	Total	151	2.63	0.92			0.508	208	3.09	0.82			**< 0.001**	0.910	
Patient-centeredness	IG	71	3.90	0.26	4.00	3.87–4.00		86	3.91	0.15	4.00	3.87–4.00			0.527
	CG	80	3.87	0.22	4.00	3.87–4.00		122	3.88	0.28	4.00	3.87–4.00			0.137
	Total	151	3.88	0.24			0.125	208	3.89	0.23			0.765	0.795	
Teamwork, roles, and responsibilities	IG	72	3.17	0.64	3.33	2.77–3.66		86	3.42	0.50	3.55	3.22–3.77			**< 0.001**
	CG	81	3.07	0.65	3.22	2.72–3.55		122	3.23	0.58	3.23	2.88–3.69			0.091
	Total	153	3.12	0.64			0.271	208	3.31	0.56			**0.012**	0.848	

*Note*: Descriptive statistics at T0 (pre training) and T1 (post training) between IG and CG and results of Wilcoxon-Test and Mann-Whitney-U-Test across the two timepoints and IG and CG for each scale

*Note*: Standard deviation (SD), Interquartile range (IR)

^a^ Wilcoxon-Test

^b^ Mann-Whitney-U-Test

Significance level *p* < .05; **bold text**: significant differences

### Feasibility aspects and implementation conditions

Five medical students and two nursing trainees were interviewed after interprofessional communication skills training about feasibility aspects and implementation conditions. Mean lengths of the interviews was 14 min and 30 s, with a range from nine to 18 min. Categories are focusing on organisation of the course (training and blended-learning format), teaching materials and the interprofessional training with the actors itself.

The organization (including preparation, course scheduling, size and lecturers) was perceived as positive. Medical students and nursing trainees felt prepared for the training. Although the teaching materials covered all the important aspects on the topic of interprofessional communication and error communication, they were also considered too extensive for the actual training.

“It was partly perhaps, not too extensive, but partly perhaps too elaborate for what you actually needed in the course.” Medical student.

In some cases, there was a reported imbalance in the distribution of professions in favor of medical students, resulting in nursing roles being trained multiple times. The interprofessional communication training on medication errors was described as very empathic and solution-oriented, with realistic situations that can strengthen interprofessional communication.

“Very, very positive. Exactly. So that was very, very interesting. Because to put yourself in the role of a registered nurse with a physician, to have this conversation with a patient. And that was, yes, quite impressive to me. And I think it’s pretty good and I would like to do it more often, just to strengthen communication and cooperation.” Nursing trainee.

Challenges were described in the agreement between the professions to deal professionally with their own and the other person’s feelings and not to let any question of guilt arise.

“But again, I found it difficult to communicate as a team. Because on the one hand I didn’t want to talk about her mistake. I wanted to let her talk about her mistake herself. But on the other hand, the patient put us in the same group.” Medical student

## Discussion

The first aim of this pilot study was the development of an interprofessional communication skills training for nursing trainees and medical students. This training should address the skill of communicating errors in an interprofessional team already during vocational training for both professions. The second aim was to test whether the interprofessional communication skills training was associated with improvements in interprofessional attitudes and self-reported skills in interprofessional error communication. Results of this pilot study support the hypothesis that both nursing trainees and medical students can be trained together, and that participants in the training show a more positive attitude towards teamwork and interprofessional error communication after training. Blended-learning format, interprofessional communication training with actors and feedback were described as positive and sustainable, although challenges were also described within the communication situations and dealing with own errors and emotions.

The IPAS is a valid and reliable instrument to measure attitudes about interprofessional learning and is used in different settings and to measure changes through IPE interventions [25–34]. When comparing the pre- and post-training results of both groups in our study, we found a significant improvement in the subscale “teamwork, roles and responsibilities” and in the self-developed interprofessional error communication scale. The improvement of attitudes regarding interprofessional teamwork after IPE is not surprising, previous studies support the benefits of IPE [35–39]. IPE interventions and trainings can lead to improvements in interprofessional teamwork, attitudes toward interprofessional teams in health care, and can help develop the skills necessary for successful improvement of patient safety topics [40–43].

We did not observe changes of the sub-scale “patient-centeredness” after training in IG and CG. However, the scale already showed high scores in both groups at T0, so that an improvement was hardly possible here. Also, the communication scenarios were developed on the basis of CIRS cases in Germany and therefore should present a realistic ADE, but were not primarily designed for testing patient-centeredness. However, one scenario deals with a conversation with an oncology patient in whom an ADE may have occurred, resulting in prolonged treatment. Aside from a statistic ceiling effect and the construction of the scenarios, another possible explanation would be the focus of the online part of the blended-learning format, which mostly refers to communication models and interprofessional error communication. Participants therefore were prepared for the training with a focus on interprofessional communication, rather than aspects of patient-centeredness. Future IPE trainings regarding error communication should take patient-centeredness outcomes more in account.

Although the utility and benefits of IPE in health professions are well-known and internationally form a standard component of the training of medical, nursing and therapeutic professionals, IPE in Germany seems to be still local projects attached to university hospitals, and there is currently no national strategy for a comprehensive implementation of (a longitudinal) IPE [44]. Future IPE studies should therefore concentrate on longitudinal courses, supporting students from different professions already from the start of their programme.

### Strengths and limitations

A major strength is the quasi-experimental pre-post study design with two study arms, through which first conclusions can be drawn about the effectiveness of the interprofessional training in comparison to regular teaching. As part of the intervention all IG participants received 360° feedback, which has become a reliable and valid tool for assessing the performance of HCPs in practice [45]. Furthermore, the integration of the interprofessional communication skills training into an existing mandatory course is a great strength, as this meant that the necessary framework was already in place for medical students and lecturers. In order to create equal conditions for nursing as well, it would be desirable to integrate communication training into the nursing curriculum. The scales used are from the G-IPAS, which has shown solid reliability [23]. Since the education focused on teamwork, patient-centredness and interprofessional error communication, we used the corresponding two scales of the G-IPAS and developed a new scale concerning the error communication. The use of a shortened version of the G-IPAS made the questionnaire short, which should increase the response rate [46].

Our study has important limitations to consider. First, because of the small sample size of IG and CG (n = 94, n = 127) and no randomisation, a sampling bias in the pilot study cannot be excluded. Due to the formal differences and the differences within the training for nursing trainees and medical students, there are considerable challenges in Germany to train both disciplines together [47]. In order to collect data from medical students and nursing trainees, there are inconsistencies in terms of practical experience due to the formal structure of both professional training programmes, which is a well described as a methodological challenge in Germany [48]. Consequently, data were collected in different ways between medical students and nursing trainees, which may have had an impact on the response rate and may have resulted in a distortion. Several strategies were used to increase the response rate: for the online questionnaire, participants were reminded weekly and the paper questionnaire was to be completed on site in person [46]. As this study is classified in the field of medical education research, the two study arms are natural groups. The quasi-experiment compares natural groups without randomized assignment of subjects. Consequently, there are differences between the data collections as well as the sample size of both study arms. In follow-up studies, data collection should be similar in the different groups. In addition, our data only describe the experience of one pilot training session in the semester, so we cannot assume that the results can be generalized. Also, we have no knowledge of an actual change in clinical practice. Therefore, the results may be valid specifically only for this national healthcare system. In order to include as large a group of nursing trainees as possible in the study, the IG and the CG of the nursing trainees were not from the same year of apprenticeship. For the medical students, the course was already firmly scheduled, which meant that time slots were already reserved in the timetable and appropriate rooms were available in the teaching building. The nursing school, on the other hand, had to determine for which nursing trainees the project would be suitable in terms of content and time. Some participants already had other courses. Time overlaps among the trainees and different attendance regulations meant that not all of the invited trainees took part in the course. It is known from the literature that different structural circumstances can have an influence on participation [48] and that different participation behavior can be a hindrance to the implementation of interprofessional events [49]. Therefore, it may be that the two groups may have different attitudes based on their knowledge and experience. Another limitation is that the conditions regarding the simulation talks of the CG were different. While the CG of the medical students received training, the CG of the nursing trainees did not. In follow-up-studies the conditions should be similar. The nursing trainees have been working in clinical care since the start of their training and already have experience in interprofessional teamwork and in dealing with errors and error communication. The medical students, on the other hand, are still at the start of their clinical study section and do not have the same level of experience. Since the interprofessional trainings were integrated into an existing course, the implementation was not possible otherwise for organisational reasons. In order to have similar conditions for the participants, the interprofessional training should take place in the long term with medical students in later phases of their training. We did not conduct an analysis of differences between medicine and nursing due to the pilot nature of the study, the associated sample size and distribution. Thus, statements about differences will need to be explored in future studies.

### Conclusions and practical implications

Self-reported skills in interprofessional error communication and attitudes regarding teamwork, roles and responsibilities improved after an interprofessional communication skills training, attended by medical students and nursing trainees. Further studies should investigate which situations arise for the communication training. Demonstrating sustainable effects of training will require longitudinal studies to capture the impact of attitudinal changes on interprofessional error communication within clinical settings.

Building on the initial results of this pilot study, it seems positive to bring together different healthcare professions already during their education. By the interviews we found that amount and content of previously provided materials should be designed in such a way that students and trainees can prepare themselves optimally but are not overwhelmed with the material. Further interviews would be necessary to make statements about the feasibility of interprofessional conversation simulation training during education.

## Data Availability

The datasets generated and/or analyzed during the current study are not publicly available but are available from the corresponding author on reasonable request.

## Abbreviations

ADE: Adverse Drug Events
CG: Control Group
CIRS: Critical Incident Reporting System
G-IPAS: German Interprofessional Attitudes Scale
HCPs: Health Care Professionals
IG: Intervention Group
IPE: Interprofessional Education
